# Construction of a Synergy Combination Model for Turmeric (*Curcuma longa* L.) and Black Pepper (*Piper nigrum* L.) Extracts: Enhanced Anticancer Activity against A549 and NCI-H292 Human Lung Cancer Cells

**DOI:** 10.3390/cimb46060332

**Published:** 2024-06-01

**Authors:** Hyun-Ki Cho, Chang-Gyun Park, Heung-Bin Lim

**Affiliations:** 1 Environmental Safety Group, Korea Institute of Science and Technology (KIST–Europe), 66123 Saarbrucken, Germany; hyunki.cho@kist-europe.de; 2 Division of Experimental Neurosurgery, Department of Neurosurgery, Heidelberg University Hospital, 69120 Heidelberg, Germany; chang.park@med.uni-heidelberg.de; 3 Department of Industrial Plant Science & Technology, Chungbuk National University, Cheongju 28644, Republic of Korea

**Keywords:** *Curcuma longa* L., *Piper nigrum* L., lung cancer cells, response surface methodology, synergistic effect

## Abstract

Extensive research on medicinal herbs for bioactive compounds proposes that they could replace synthetic drugs, reducing side effects and economic burdens. Especially, interest in the synergistic benefits of natural products is increasing, implying that their combined use may enhance therapeutic effectiveness. This study aimed to explore the synergetic effects of turmeric (*Curcuma longa* L.) and black pepper (*Piper nigrum* L.) extract on lung normal (MRC-5) and cancer (A549 and NCI-H292) cell lines. The turmeric extract (TM) only affected the lung cancer cell lines, but it had no impact on the MRC-5 cell line. On the other hand, the black pepper extract (BP) did not cause any damage to either the lung normal or cancer cell lines, even at concentrations of up to 400 µg/mL. Response surface methodology was used to predict the ideal synergistic concentrations (EC_50_) of TM and BP, which were found to be 48.5 and 241.7 µg/mL, respectively. Notably, the selected condition resulted in higher cytotoxicity compared to the exposure to TM alone, indicating a potent synergetic effect. The rate of curcumin degradation under this combined treatment was significantly decreased to 49.72 ± 5.00 nmol/h/µg for A549 cells and 47.53 ± 4.78 nmol/h/µg for NCI-H292 cells, respectively, as compared to curcumin alone. Taken together, this study confirmed the potent synergistic effect of TM and BP on lung cancer cell lines. Further research is required to identify their specific synergetic mechanisms. Our findings provide crucial foundational data on the synergistic effects of TM and BP.

## 1. Introduction

Lung cancer is a major cause of death around the world, exceeding mortality rates from colon, breast, and prostate cancers [1]. About 70% of lung cancer patients die due to the aggressive metastatic spread of the disease [2]. Although chemotherapy is a widely used treatment, it also affects healthy tissues [3]. Therefore, choosing the right therapeutic agents that provide high efficacy while minimizing side effects is crucial in managing lung cancer. Meanwhile, there has been a significant increase in research over a few decades that aims to explore the potential of natural products for the development of new materials. The substances offer a promising alternative to synthetic drugs, which can be expensive and often have more negative side effects [4]. Natural compounds, such as curcumin, piperine, resveratrol, lycopene, and sulforaphane, reveal a broad range of physiological benefits, including anti-cancer properties in different types of cells [5]. These agents are highly effective and have minimal side effects, making them valuable complementary treatments in cancer therapy [4,5]. Among these, curcumin is a primary polyphenol found in turmeric (*Curcuma longa* L.) and has many beneficial properties, such as anti-aging, anti-inflammatory, choleretic, anti-ulcer, and anti-tumor effects [6,7]. It is also non-toxic to both humans and animals, which is why the American Food and Drug Administration (FDA) has recognized it as “Generally Recognized as Safe” (GRAS) [8]. However, its effectiveness is limited due to its low absorption rate and swift metabolism [9]. To address this issue, co-administration with adjuvants has been suggested as a strategy to enhance its therapeutic potency [9,10]. Among various adjuvants, piperine is known for its ability to boost curcumin’s bioavailability by inhibiting enzymes responsible for metabolism processes [9,11,12]. Piperine is a primary active component in black pepper (*Piper nigrum* L) and can slow down the metabolism of drugs and improve their bioavailability, making them more effective [11,13,14]. For these reasons, combining curcumin with piperine has been found to surpass the efficacy of curcumin alone. The combined effects of curcumin and piperine slow down curcumin’s metabolic degradation and improve its absorption [13,14].

Herein, our study aims to investigate the selective cytotoxic effects of the combination of turmeric (TM) and black pepper (BP) extracts on lung cancer cells. The results demonstrated that the combination of TM and BP extracts was more effective in reducing cancer cell viability compared to TM extract alone, indicating a synergetic effect. Consequently, our study provides insightful information on the synergistic effects of TM and BP for lung cancer therapeutic effectiveness.

## 2. Materials and Methods

### 2.1. Chemicals

Dimethyl sulfoxide (DMSO), curcumin, piperine, monopotassium phosphate, dipotassium phosphate, phosphoric acid, and neutral red were acquired from Sigma-Aldrich Corporation (St. Louis, MO, USA). Phosphate-buffered saline (PBS, pH 7.4), fetal bovine serum (FBS), L-glutamine, penicillin/streptomycin mix, Roswell Park Memorial Institute (RPMI) 1640 culture medium, and trypsin were sourced from GIBCO (Grand Island, NY, USA). All additional chemicals and reagents utilized were of analytical quality.

### 2.2. Extraction of Curcumin and Piperine

Fresh TM and BP were sourced from a farm in Jindo, Republic of Korea, as powder, and from Lebensbaum in Diepholz, Germany, respectively. The dried TM and BP were finely ground, and these ground samples were extracted with 70% ethanol (*v*/*v*, 800 mL) in a Soxhlet extractor for 24 h at 90 °C. Following the extraction phase, the resultant solutions were condensed under reduced pressure using a rotary evaporator (Eyela, Tokyo, Japan) and then freeze-dried utilizing a lab-scale freeze dryer (ilShinBioBase Co., Ltd., Dongducheon, Republic of Korea).

### 2.3. Component Analysis

To measure the concentrations of curcumin and piperine, the freeze-dried extracts from TM and BP were dissolved in methanol. These preparations were then subjected to analysis using the Agilent 1100 series high-performance liquid chromatography (HPLC) apparatus, equipped with a binary solvent delivery system, an automatic sample injector, and a multi-wavelength detector (MWD). The chromatographic separation for curcumin was executed on a Shiseido Capcell Pak C18 column (4.6 mm × 500 mm, 5 μm). The mobile phase solution consisted of a 25 mM potassium dihydrogen orthophosphate buffer (pH 3.5) (solvent A) and acetonitrile (solvent B). Detection and quantification of curcumin and piperine were achieved with the MWD set at wavelengths of 421.5 nm for curcumin and 342.5 nm for piperine, respectively.

### 2.4. Cell Culture and Viability

The A549, NCI-H292 (alveolar type-II carcinoma and mucoepidermoid carcinoma), and MRC-5 (normal lung fibroblast) cell lines were acquired from the Korean Cell Line Bank (KCLB). The cells were cultured in RPMI 1640 and MEM media, respectively, both supplemented with 10% FBS, 1% penicillin and streptomycin. The cell lines were cultivated in a moisture-controlled incubator (5% CO_2_ and 95% air) at 37 °C. The cell lines were sub-cultured every 2–3 days.

The neutral red uptake assay was used for assessing cell viability and the assay was conducted following the method in [15]. Briefly, cells were distributed into 96-well plates at a concentration of 1.0 × 10^4^ cells per well. The cells were incubated for 24 h at 37 °C in an environment consisting of 95% air and 5% CO_2_. Subsequently, the original medium was removed from the wells and replaced with media containing the samples. After 24 h, the medium used for treatment/control was substituted with 200 μL of neutral red dye solution (50 μg/mL in FBS-free culture medium). After a 3-h incubation, the dye solution was withdrawn, and to each well was added 200 μL of a wash/fixative solution (1% formaldehyde) for 1 min at room temperature. This solution was promptly removed, and 200 μL of a neutral red desorption solution (1% acetic acid in 50% ethanol) was added to each well. The plates were then agitated on a microplate shaker for 10 min. The absorbance for each well was measured at 540 nm using a microplate reader (Thermo Scientific, Vantaa, Finland).

### 2.5. Response Surface Methodology

#### 2.5.1. Central Composite Design

The central composite design, a fundamental approach within response surface methodology, was employed to determine the optimal combination of TM and BP extracts on the test cells. Design-Expert version 11 software (Stat-Ease Inc., Minneapolis, MN, USA) was used to analyze data and estimate regression equation coefficients [16]. The concentrations of TM and BP extracts (*X*_1_ and *X*_2_) were set as the independent variables employed in the central composite design. Each variable was tested at five levels (TM extract: 10, 20, 30, 40, and 50 μg/mL; BP extract: 200, 250, 300, 350, and 400 μg/mL).

#### 2.5.2. Model Construction

After the central composite design, the model construction was implemented to accurately predict the response behavior as the input variables changed. To achieve this, a quadratic polynomial model was applied to capture both the curvature and interactions between the variables. The equation of the quadratic response model was as follows:

(1) $$Y=\beta_{0}+\sum_{i=1}^{2}\beta_{i}X_{i}+\sum_{i=1}^{2}\beta_{ii}X_{i}^{2}+\sum_{i=1}^{1}\sum_{j=i+1}^{2}\beta_{ij}X_{i}X_{j}$$

where *Y* represents the response variable measured as cell viability (%). *X_i_* and *X_j_* denote the model’s independent variables. The coefficients *β*_0_, *β_i_*, *β_ii_*, and *β_ij_* correspond to the intercept, linear, quadratic, and interaction terms, respectively, within the response surface equation. Then, coefficients were estimated through least squares regression, which minimizes the sum of the squared errors between observed outcomes and those predicted values by the model (Supplementary Table S2).

#### 2.5.3. Desirability Function

After the model construction, the desirability function was applied to optimize combination concentration. The optimal concentration of both extracts at the EC_50_ value was determined using a desirability function approach. The method transforms each predicted response into a dimensionless desirability value (*d_i_*), ranging from 0 to 1. A value of 0 indicates an undesirable outcome, which is outside the acceptable range. On the other hand, a value of 1 represents the most desirable outcome, achieving the goal. The value was computed based on the specific goals for each response variable, including maximization, minimization, and hitting a target value. Then, the overall desirability score (*D*) is computed by taking the geometric mean of the *d_i_* values. The formulation is given as follows:

(2) $$D=(d_{1}\times d_{2}\times d_{3}\times \cdots \times d_{n}{)}^{1/n}={\left(\prod_{i=1}^{n}d_{i}\right)}^{1/n}$$

where *n* is the number of responses in the measure. After computing the optimal conditions using the desirability function, the conditions were tested in the synergistic experiment to confirm whether the predicted results were achievable in practice.

### 2.6. Synergistic Effect

#### 2.6.1. Combination Index

To verify the synergistic effect, a combination index (CI) model analysis was employed. Cell viability data obtained from simultaneous exposure to TM and BP were inputted into CompuSyn software version 2.0 (Biosoft, Cambridge, UK). The software was used to generate a plot of the combination index-fraction affected (CI-Fa) [17]. A CI value indicates synergy (CI < 1), an additive effect (CI = 1), or antagonism (CI > 1).

#### 2.6.2. Curcumin Consumption Activity

In addition, the consumption activity was conducted to evaluate the curcumin uptake activity. A549 and NCI-H292 lung cancer cells were cultured in 60 mm dishes, each seeded with 3.0 × 10^5^ cells. These dishes were incubated for 24 h at 37 °C within an environment containing 95% air and 5% CO_2_. Afterward, the original medium was removed and replaced with media containing TM and BP, diluted to concentrations of 48.5 and 241.7 μg/mL, respectively, for durations of 2, 4, 6, 8, and 10 h. The sample preparation followed the method outlined by Schiborr et al. [18]. 500 μL of each diluted sample was transferred into test tubes and vortexed for 20 s. Then, 900 μL of a solvent mixture (EtOH/MeOH, 95:5 ratio) was added to reach a total volume of 1.4 mL. After centrifuging at 20,000× *g* for 15 min at 4 °C, the samples were processed using a Waters Acquity ultra performance liquid chromatography (UPLC) system (Waters Corp., Milford, MA, USA), equipped with a binary solvent pump, autosampler, and photodiode array (PDA) detector. Curcumin was separated on a Waters ACQUITY UPLC BEH C18 column (2.1 × 50 mm, 1.7 μm) with a mobile phase of 0.1% formic acid in water (Solvent A) and acetonitrile (Solvent B), a column temperature of 40 °C, a flow rate of 0.3 mL/min, and an injection volume of 1 μL. The elution gradient was from 10% to 100% B over 5 min, then back to 10% B, with a total run time of 7 min. The curcumin consumption rate (*σCC*, nmol/h) was calculated using a simplified linear regression from the concentration over time [19]. The linear regression is given as follows:

(3) $$\sigma CC=-\sigma Abs\times \frac{N_{0}}{{Abs}_{0}}$$

where *σAbs* represents the slope of concentration over time; *Abs*_0_ indicates the initial concentration calculated from the linear regression of concentration over time; *N*_0_ represents the initial curcumin moles in media diluted with TM (48.5 μg/mL) and BP (241.7 μg/mL). The curcumin consumption activity was determined by first deducting the blank values (bTM, without cells) from the *σCC* of the samples (TM and TMBP, containing cells). This result was then normalized to the initial mass of curcumin present in the media diluted with TM and BP, as follows:

(4) $$CC\;activity=\frac{\sigma CC-{\sigma CC}_{black}}{{CC}_{mass}}$$

The mass-normalized curcumin consumption activity (*CC activity*) value serves as a comparative measure of the rate at which curcumin is consumed over time, starting from the initial point of reaction.

### 2.7. Statistical Analysis

The outcomes of the toxicology studies were presented as mean ± standard deviation. To analyze the collected data, Analysis of Variance (ANOVA) was employed for evaluating the differences across all measured values. For comparisons between two groups, Tukey’s test was utilized. A *p*-value of less than 0.05 was deemed to indicate statistical significance. All statistical analyses were conducted using SPSS Statistics 24 for Windows (IBM Corp., Armonk, NY, USA).

## 3. Results and Discussion

### 3.1. Cytotoxicity

**Figure 1:**
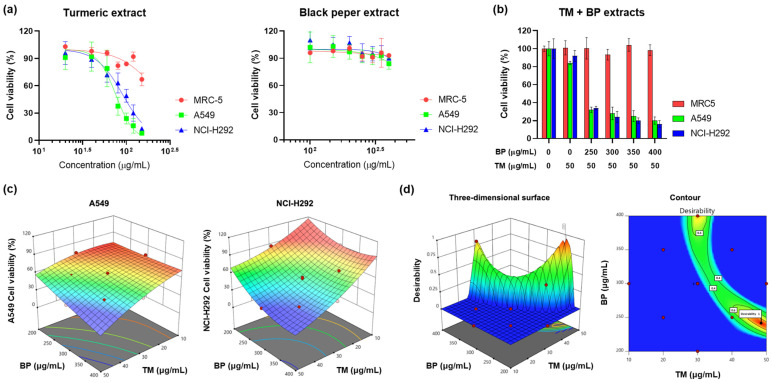
Cytotoxicity assessment and response surface analysis of turmeric (TM) and black pepper (BP) extract. (a) The cells were treated with TM and BP extracts (μg/mL) for 24 h, respectively. Data are expressed as mean ± SD of three repeated experiments. (b) The cytotoxicity assessment of the co-exposure TM and BP extracts. Data are expressed as mean ± SD of three repeated experiments. (c) Three-dimensional response surface plots of the cell viability of A549 and NCI-H292, TM, and BP extracts. (d) Response surface graph and contour plots of optimal desirability function at the EC_50_ value on A549 and NCI-H292 lung cancer cell lines.

The yields of TM and BP extracts were approximately 18.2% *w*/*w* and 8.2% *w*/*w*, respectively. High performance liquid chromatography (HPLC) was used for analysis, and the results are shown in Supplementary Figure S1. The content of curcumin in the TM extract and piperine in the BP extract were 26.7 ± 3.1 and 153.1 ± 7.4 μg/mg, respectively, which are higher than the ranges reported in other studies [20,21]. The extracts were then applied to the cell lines as presented in Figure 1a. TM caused significant cytotoxic effects on the lung cancer cell lines. The cytotoxic effect was only observed at the highest concentration (150 μg/mL) tested on the MRC-5 cell line. The EC_50_ values for A549 and NCI-H292 cancer cells were 77.8 and 92.0 μg/mL, respectively. In contrast, black pepper extract (BP) did not induce significant cytotoxicity up to a concentration of 400 μg/mL for any of the three cell types tested. To explore the potential synergistic effects of combining TM and BP, the combined extracts were administered to MRC-5, A549, and NCI-H292 lung cancer cells at concentrations where no significant reduction in cell viability was observed, as illustrated in Figure 1b. The combinations of TM (50 μg/mL) and BP (250–400 μg/mL) induced potent cytotoxic effects on both A549 and NCI-H292 lung cancer cell lines. Interestingly, these combinations did not significantly decrease cell viability in MRC-5 cells. This selective cytotoxicity towards lung cancer cells is likely to be related to the function of curcumin, which has been reported to possess higher specificity for cancer cells over normal cells by activating various cellular mechanisms that are more commonly altered in cancerous tissues [22]. Curcumin targets pathways upregulated in cancer cells, enhancing its therapeutic potential without affecting normal cells [23]. These target pathways, such as transcription factor downregulation, apoptosis sensitization, folate conjugation, and ROS generation, collectively contribute to acting more specifically on cancer cells, making it a potential targeted therapy for cancer treatment [24,25,26]. On the other hand, BP did not show significant toxicity in all cells at concentrations up to 400 μg/mL. This is consistent with the previous study [27]. The BP extract exhibited only 8% cell death at 300 μg/mL. Through the cytotoxicity test, we confirmed that the TM extract has selective cytotoxicity against lung cancer cells and shows potent synergistic cytotoxicity in the presence of the BP extract. 

### 3.2. Response Surface Analysis

Response surface analysis was conducted to predict and select the most effective condition of the TM and BP combination based on cytotoxicity results [28]. For the analysis, a central composite design was performed to identify ideal conditions in complex experimental setups (Supplementary Tables S1 and S2). The central composite design involved 14 operation conditions (*X*_1_ and *X*_2_), and the cell viability response. The results showed that both model terms *X*_1_ and *X*_2_ significantly impacted the model outcomes. The computed values, such as the Lack of Fit, adjusted determination coefficient, and Adeq Precision, indicated that the predictive ability of the current model is reasonable and the polynomial equation derived from the analysis is valid. Supplementary Tables S1 and S2 provide detail information on the central composite design. Three-dimensional response surface plots showed that the cytotoxicity of the cancer cells depended on the concentrations of TM and BP combinations, as shown in Figure 1c. In both models, cell viability decreased with increasing concentrations of the extracts. Using a desirability function approach based on the central composite design, the optimal combination ratio of TM and BP extracts for the EC_50_ value was determined and the desirability of the response surface is depicted in Figure 1d and Table 1. Acceptable and reliable desirability values were computed for all five solutions. The condition at the highest value was 48.5 and 241.7 μg/mL for TM and BP, respectively. Notably, the coefficient of variation between the actual and optimized cell viability for A549 and NCI-H292 lung cancer cells was less than 10%, confirming the predictive accuracy. Although A549 and NCI-H292 cells exhibited different viability responses to TM and BP, a single optimal concentration was calculated to reflect the combined response values from both cell types. The experimental results closely matched the theoretical predictions, as indicated by the uniformity of the desirability values across the board (Table 1). A desirability value of 1 represents the optimal combination concentration for achieving the EC_50_ value, which was subsequently used in further experiments.

### 3.3. Synergistic Effect and Consumption Activity

**Figure 2:**
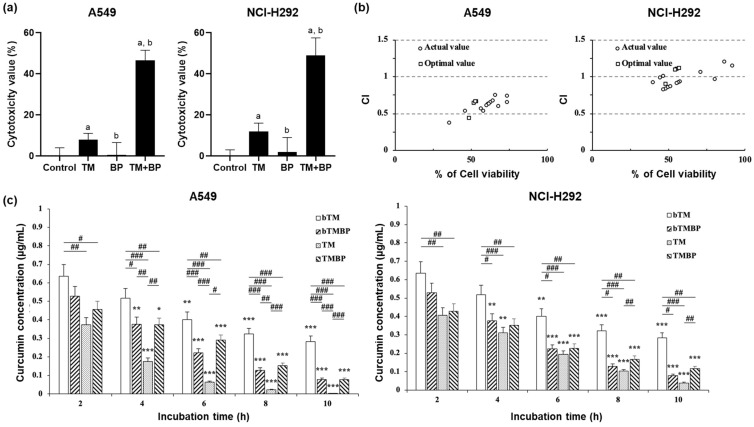
Synergistic effect and curcumin consumption activity. (**a**) The synergistic effect of the TM and BP combination at the EC_50_ value. Data are expressed as mean ± SD of three repeated experiments. The letter ‘a’ indicates a significant difference from control; ‘b’ indicates a significant difference from TM. (**b**) Combination index (CI) plots of the combination of TM and BP on A549 and NCI-H292 lung cancer cell lines. Previous combination data are the cytotoxicity results in Figure 1b. Optimal combination data are the cytotoxicity results of the optimal combination of both extracts at the EC_50_ value. (**c**) Curcumin consumption rate with/without A549 and NCI-H292 cell lines. Data are expressed as mean ± SD of three repeated experiments. * *p* < 0.05, ** *p* < 0.01 and *** *p* < 0.005 when groups were compared with the 2 h group, and # *p* < 0.05, ## *p* < 0.01 and ### *p* < 0.001 when compared within time-groups were significantly different according to one-way analysis of variance (ANOVA) with Tukey’s multiple comparison tests. bTM, turmeric extract incubated medium without cells; TM, turmeric extract incubated medium with cells; TMBP, turmeric plus black pepper extracts-incubated medium with cells.

According to the condition of the highest desirability value, the synergistic effect of the combination of TM and BP extract was investigated on the lung cancer cell lines (Figure 2a). Of note, the combination of TM and BP extracts caused potent cytotoxic effects for both cancer cell lines compared to their single exposure. Cytotoxicity values of the TM and BP combination in A549 and NCI-H292 lung cancer cell lines were 52.4% and 51.4%, respectively. The values were 3−4 times higher than in the single exposure. To define the synergistic effect, the combination index (CI) was calculated (Figure 2b). The concept of synergism is quantitatively defined by synergism (CI < 1), additive effect (CI = 1), and antagonism (CI > 1) [17]. The combination showed synergistic interactions in both the previous and optimal combinations for both cell lines. This was indicated by the CI values being less than 1 across all tested concentrations. The previous combinations are the results of the cytotoxic effect, as shown in Figure 1b. After the confirmation of the synergistic effect, we investigated the curcumin consumption activity to understand synergistic mechanisms. The activity (nmol/h/μg) was measured on both lung cancer cell lines at 2, 4, 6, 8, and 10 h, respectively (Figure 2c). Interestingly, the consumption rate of the TM and BP combination exhibited a lower consumption rate compared to that of the TM single exposure. This phenomenon was observed in both lung cancer cell lines. The rates for the single TM exposure on A549 and NCI-H292 were 116.14 ± and 76.02 ± 7.64 nmol/h/μg, respectively, whereas the consumption rate of the TM and BP combination on A549 and NCI-H292 cell lines was 49.72 ± 5.00 and 47.53 ± 4.78 nmol/h/μg, respectively, suggesting that the BP extract interfere curcumin metabolisms. 

In this study, we confirmed the potent synergistic cytotoxic effect in the TM and BP combination. Considering the content of curcumin and piperine in both extracts and the most desirable condition, it seems that the synergistic cytotoxic effect of turmeric and black pepper extracts is mainly caused by the combination of curcumin and piperine. Both compounds are major components in the extracts and have been shown to have a synergistic effect in previous studies [10,12,13,14]. However, other components, such as essential oils, polyphenols, and alkaloids, in the extracts might also impact the cancer cells. These compounds extracted from other plants revealed potent cytotoxic effects on cancer cells in the previous study [4,29,30]. Therefore, it will be also meaningful to conduct future studies that focus on identifying the effects of other components present in both extracts. Of note, we observed the delayed curcumin consumption rate in the TM and BP combination. The delayed consumption rate seems closely linked to the improved bioavailability of curcumin. Banji et al. reported that piperine improved the bioavailability of curcumin by changing the fluidity of the brush border membrane and controlling cell dynamics [31]. Khajuria et al. demonstrated that piperine can enhance the bioavailability by modifying membrane lipid dynamics and enzyme form, increasing residence time, and attenuating metabolic rate [32]. In addition, piperine inhibited the function of the P-glycoprotein (ABCB1 or MDR1) efflux pump, which increases the bioavailability of curcumin [33,34]. Taken together, the TM and BP combination can lead to a synergistic effect that alters cell dynamics due to increased curcumin residence time caused by piperine activation.

## 4. Conclusions

In this study, we confirmed the potent synergistic effects of combining extracts of TM and BP on lung cancer cell lines, while having no impact on normal lung cells. The TM extract selectively targeted cancer cells, while the BP extract was non-toxic across all tested cell lines, even at the highest concentration. By using response surface methodology, we identified the optimal synergistic concentrations for TM and BP extracts, which significantly enhanced cytotoxicity against lung cancer cells compared to TM alone. Moreover, the combination treatment notably slowed the rate of curcumin degradation, further indicating the therapeutic potential of these natural extracts when used together. The current study highlights the development of a synergy combination model for TM and BP, which significantly enhances the therapeutic effectiveness against cancer cells while minimizing the impact on normal cells. These findings suggest that TM and BP extracts could provide a foundation for developing safer and more effective therapeutic options for lung cancer. Further research is needed to clarify the specific mechanisms behind their synergistic effects, which could guide the future of natural product-based therapies in oncology.

## Figures and Tables

**Table 1:** The validation of the top five ranked predicted values of optimal combinations according to the desirability values.

Rank	Desirability Value	Concentration (μg/mL)		Predicted Value (%)		Experimental Value (%)	
		Turmeric	Black Pepper	A549	NCI-H292	A549	NCI-H292
1	1	48.5	241.7	50	50	47.6 ± 5.9	48.6 ± 6.2
2	0.816	29.7	400	53.3	49.9	53.1 ± 6.1	52.3 ± 4.5
3	0.816	29.6	400	53.3	50	51.6 ± 5.6	52.2 ± 4.4
4	0.815	29.7	400	53.2	49.8	52.5 ± 3.8	51.8 ± 3.9
5	0.814	29.8	400	53.1	49.6	52.1 ± 5.3	51.5 ± 5.3

Data are expressed as mean ± SD of three repeated experiments.

## Data Availability

The data presented in this study are available on request from the corresponding author. The data are not publicly available due to reasons of privacy.

## Supplementary Materials

The following supporting information can be downloaded at: https://www.mdpi.com/article/10.3390/cimb46060332/s1, Figure S1: Representative High performance liquid chromatography with multiple wavelength detector (HPLC-MWD; 421.5 nm and 342.5 nm) chromatogram of curcumin and piperine in TM and BP, respectively (0.4 ug/mL); Table S1: Central composite design and cell viability for turmeric and black pepper extracts; Table S2: Analysis of variance for central composite design and test results of the regression coefficients and intercepts of coded equations for cell viability.
