# Supplementary material for: Construction of a Synergy Combination Model for Turmeric (Curcuma longa L.) and Black Pepper (Piper nigrum L.) Extracts: Enhanced Anticancer Activity against A549 and NCI-H292 Human Lung Cancer Cells
Source: Curr Issues Mol Biol. 2024 Jun 1;46(6):5551–60. doi: 10.3390/cimb46060332 (PMC11201915; doi:10.3390/cimb46060332)
Supplement: Supplementary file 1 [file cimb-46-00332-s001.zip › cimb-2988713-supplementary.pdf]

Supplementary Materials

Construction of a Synergy Combination Model for Tur-meric (*Curcuma longa* L.) and Black Pepper (*Piper nigrum* L.) Extracts: Enhanced Anticancer Activity against A549 and NCI-H292 Human Lung Cancer Cells

Hyun-Ki Cho [1,†], Chang-Gyun Park [2,†] and Heung-Bin Lim [3,*]

[1] *Environmental Safety Group, Korea Institute of Science and Technology (KIST–Europe), Saarbrucken, Germany; hyunki.cho@kist-europe.de*

[2] *Division of Experimental Neurosurgery, Department of Neurosurgery, Heidelberg University Hospital, Hei-delberg, Germany; chang.park@med.uni-heidelberg.de*

[3] *Department of Industrial Plant Science & Technology, Chungbuk National University, Cheongju, Republic of Korea; heungbin@chungbuk.ac.kr*

[†] These authors contributed equally to this work.

[*]**Corresponding Authors**:

Heung-Bin Lim; E-mail address: *heungbin@chungbuk.ac.kr*;

Tel: +82-43-261-2521

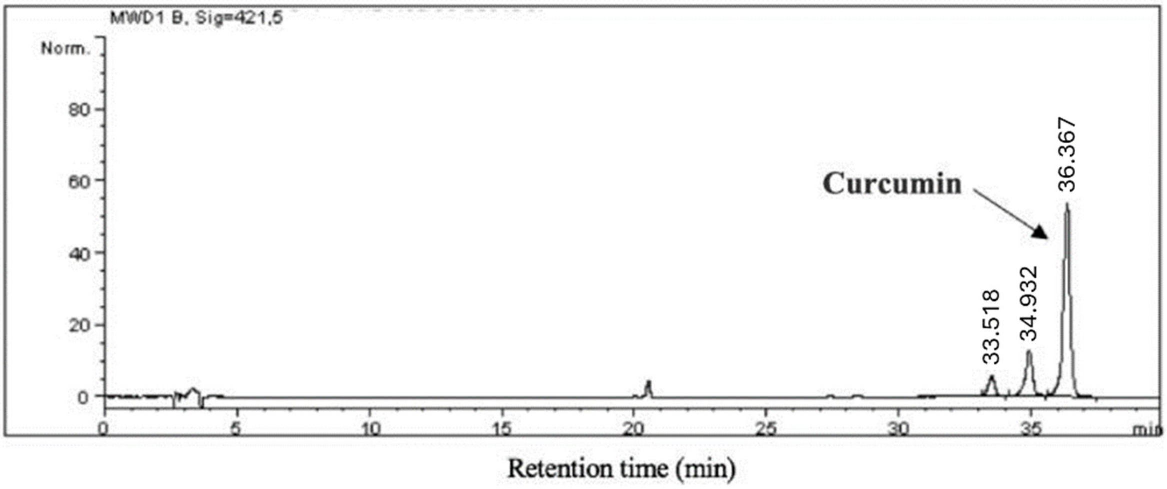

A1

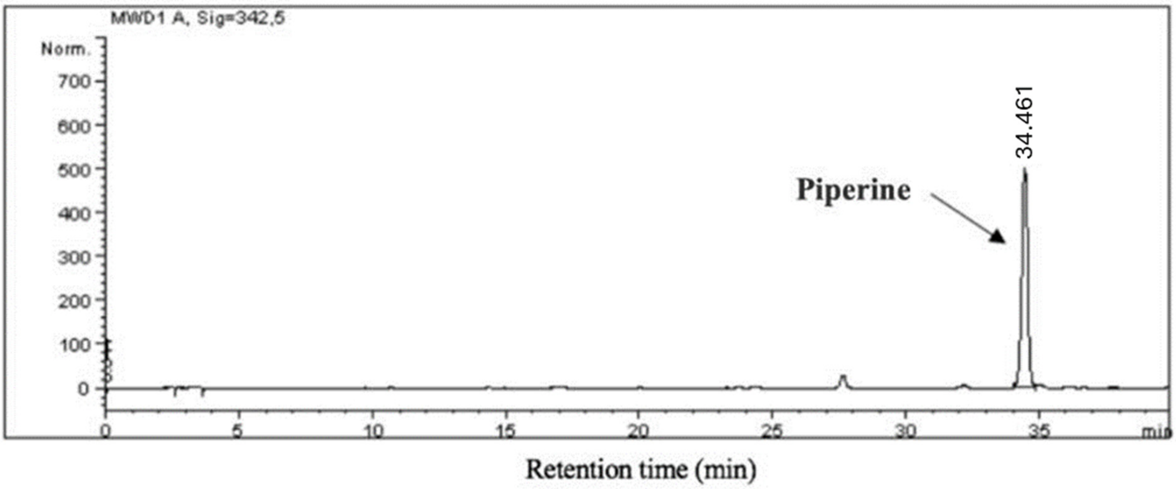

A2

Supplementary Figure S1. Representative High performance liquid chromatography with multiple wavelength detector (HPLC-MWD; 421.5 nm and 342.5 nm) chromatogram of curcumin and piperine (A1 and A2) in TM and BP, respectively (0.4 ug/mL).

Supplementary Table S1. Central composite design and cell viability for turmeric and black pepper extracts.

Runs	Factors		Cell viability (%)	
	X_1	X_2	A549	NCI-H292
1	30	300	61	49.8
2	20	350	65.6	70.8
3	20	250	67.9	80
4	40	350	46.2	34.5
5	30	300	62.8	46.4
6	40	400	58.1	51.3
7	30	300	56.4	56.4
8	30	400	52.1	46.5
9	30	300	60.1	48.1
10	50	300	35.5	29.6
11	30	300	61.6	57.5
12	30	200	73.4	86.6
13	30	300	63.6	55.3
14	10	300	73.4	91.6

X_1 and X_2, concentration (μg/mL) of turmeric and black pepper extracts, respectively.

Supplementary Table S2. Analysis of variance for central composite design and test results of the regression coefficients and intercepts of coded equations for cell viability.

Source		Mean squares	F	P values	Coefficient estimates
Cell viability	Intercept				61.13
(A549)	X_1	918.75	130.45	0.0001	
	X_2	268.85	38.17	0.0005	
	X_1X_2	23.04	3.27	0.1134	
	X_1^1	70.94	10.07	0.0156	
	X_2^2	1.91	0.2712	0.6186	
	Lack of Fit	7.99	1.26	0.3998	
	R^2 = 0.9373	Q2 = 0.7525	Adeq Precision = 18.7934		
Cell viability	Intercept				52.57
(NCI-H292)	X_1	2976.75	138.51	0.0001	
	X_2	939.87	43.73	0.0003	
	X_1X_2	14.44	0.6719	0.4394	
	X_1^1	99.36	4.62	0.0686	
	X_2^2	288.87	13.44	0.008	
	Lack of Fit	15.03	0.5709	0.6634	
	R^2 = 0.9373	Q2 = 0.8427	Adeq Precision = 19.2190		

X_1 and X_2, concentration (μg/mL) of turmeric and black pepper extracts, respectively.
The following polynomial equations can be regressed:

$Y_{cell\ viability\ (A549)} = 67.072 + 1.635X_1 – 0.021X_2 – 0.005X_1X_2 – 0.018X_1^2 + 10^{-4}X_2^2$

$Y_{cell\ viability\ (NCI\text{-}H292)} = 267.197 - 1.701X_1 – 0.926X_2 – 0.004X_1X_2 – 0.021X_1^2 + 0.001X_2^2$
